# Early life stress induces dysregulation of the heme pathway in adult mice

**DOI:** 10.14814/phy2.14844

**Published:** 2021-05-27

**Authors:** Yasminye D. Pettway, Thomas H. Neder, Dao H. Ho, Brandon M. Fox, Mariah Burch, Jackson Colson, Xiaofen Liu, Cailin E. Kellum, Kelly A. Hyndman, Jennifer S. Pollock

**Affiliations:** ^1^ Cardio‐Renal Physiology and Medicine Division of Nephrology Department of Medicine University of Alabama at Birmingham Birmingham Alabama USA

**Keywords:** early life stress, haptoglobin, heme, hemoglobin, maternal separation with early weaning, superoxide

## Abstract

Early life stress (ELS) is associated with cardiovascular disease (CVD) risk in adulthood, but the underlying vascular mechanisms are poorly understood. Increased hemoglobin and heme have recently been implicated to mediate endothelial dysfunction in several vascular diseases. Chronic physiological stress is associated with alterations in the heme pathway that have been well‐described in the literature. However, very little is known about the heme pathway with exposure to ELS or chronic psychosocial stress. Utilizing a mouse model of ELS, maternal separation with early weaning (MSEW), we previously reported that MSEW induces endothelial dysfunction via increased superoxide production. We reasoned that heme dysregulation may be one of the culprits induced by MSEW and sustained throughout adulthood; thus, we hypothesized that MSEW induces heme dysfunction. We investigated whether circulating levels of heme, a circulating pro‐oxidant mediator, are increased by MSEW and examined the role of the heme metabolic pathway and heme homeostasis in this process. We found that circulating levels of heme are increased in mice exposed to MSEW and that plasma from MSEW mice stimulated higher superoxide production in cultured mouse aortic endothelial cells (MAECs) compared to plasma from normally reared mice. The heme scavenger hemopexin blunted this enhanced superoxide production. Splenic haptoglobin abundance was significantly lower and hemoglobin levels per red blood cell were significantly higher in MSEW versus control mice. These findings lead us to propose that ELS induces increased circulating heme through dysregulation of the haptoglobin‐hemoglobin system representing a mechanistic link between ELS and CVD risk in adulthood.

## Graphical abstract

Early life stress (ELS) is associated with cardiovascular disease (CVD) risk in adulthood, but the underlying vascular mechanisms are poorly understood. Increased hemoglobin and heme have recently been implicated to mediate endothelial dysfunction in several vascular diseases. However, very little is known about the heme pathway with exposure to ELS or chronic psychosocial stress. We found that ELS induces increased circulating heme through dysregulation of the haptoglobin‐hemoglobin system representing a mechanistic link between ELS and CVD risk in adulthood.

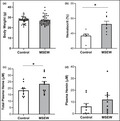

## 1 INTRODUCTION

Recent estimates predict that cardiovascular disease (CVD) affects over one‐third of the U.S. population and is a leading cause of mortality worldwide (Mozaffarian et al., 2015; Roth et al., 2015; Virani et al., 2020). A known independent risk factor for CVD is exposure to adverse childhood experiences (ACEs), also collectively referred to as early life stress (ELS) (Danese et al., 2009). ELS or ACEs are considered a chronic psychosocial stressor with exposure prior to age 18 and are well‐described to be a risk factor for depression and anxiety in adults (Merrick et al., 2017). Examples of ACEs include household dysfunction, neglect, physical abuse, and sexual abuse. ACEs are common in many countries worldwide (Kessler et al., 2010). Approximately 60 percent of U.S. adults report experiencing at least one or more ACEs, with 30 percent experiencing three or more ACEs, a statistic that has remained consistent to the present day since the original study conducted by the CDC and Kaiser Permanente in the 1990 s (CDC, 2019; Felitti et al., 1998).

Early life stress represents a highly prevalent CVD risk factor and thus a deeper mechanistic understanding is required to address this problem. Clinical and epidemiological studies have demonstrated that increased exposure to ACEs correlates with an upward trajectory of blood pressure, higher aortic pulse wave velocity, vascular dysfunction, and systemic inflammation in adulthood which are all factors that may work together to promote vascular disease (Danese et al., 2007; Rafiq et al., 2020; Su et al., 2014, 2015). Rodent models of ELS, such as the mouse model utilized in this study, all demonstrate anxiety and depression in adulthood and have begun to provide insight into the role of ELS in CVD (Ho et al., 2016; Loria et al., 2014; Murphy et al., 2017; Obi et al., 2019).

Heme‐containing proteins play essential roles in a diverse range of physiological processes, including oxygen transport, metabolism, electron transport, and cell signaling (Nagababu & Rifkind, 2004; Sawicki et al., 2015). However, excess heme and hemoglobin have been shown to be detrimental in neuronal and vascular cell function (Chiabrando et al., 2018, Jeney et al., 2002) inducing red blood cell hemolysis, cytokine upregulation, inflammation, and reactive oxygen species (ROS) production resulting in potential endothelial dysfunction (Gladwin & Kato, 2007; Graca‐Souza et al., 2002; Maio et al., 2011; Porto et al., 2007; Sawicki et al., 2015). We reasoned that excess heme may be one of the key mediators induced and sustained in adulthood with exposure to ELS. Thus, we hypothesized that ELS exposure induces excess heme and heme metabolism dysfunction in adult mice.

Enhanced TLR4 activity with heme activation is implicated in vascular dysfunction and the progression of CVD (Belcher et al., 2014; Frantz et al., 2007; Hernanz et al., 2015; Janciauskiene et al., 2020). Activation of TLR4 triggers a signaling cascade leading to superoxide production and upregulation of pro‐inflammatory cytokines (Belcher et al., 2014; Frantz et al., 2007; Janciauskiene et al., 2020; Nakashima et al., 2015; Park et al., 2004). We recently reported increased TLR4 protein abundance and inflammation in the kidneys of rats exposed to maternal separation indicating that ELS induces immune priming in the kidney (De Miguel et al., 2018). Interestingly, unfavorable childhood socioeconomic status, a source of ELS, was associated with increased TLR4 expression in peripheral blood leukocytes during adolescence (Miller & Chen, 2007). This evidence suggests a role for TLR4 in the origin or progression of ELS‐induced CVD. Thus, we also hypothesized that ELS exposure upregulates endothelial TLR4 abundance in adult mice.

The purpose of the present study was to test the hypothesis that ELS exposure induces dysfunction in the heme pathway and/or increases endothelial TLR4 abundance in adult mice compared to normally reared control mice. We investigated whether plasma levels of heme, a circulating pro‐oxidant mediator, are increased in a mouse model of ELS, maternal separation with early weaning (MSEW), and examined the role of the heme metabolic pathway and heme homeostasis in this process. Further, we evaluated aortic TLR4 gene expression, protein abundance and localization in aortic segments from adult male mice exposed to MSEW compared to normally reared control mice.

## 2 METHODS

### 2.1 Mouse model of early life stress: Maternal Separation with Early Weaning (MSEW)

All studies were approved by the Institutional Animal Care and Use Committee at the University of Alabama at Birmingham (UAB) and conducted according to the *Guide for the Care and Use of Laboratory* Animals provided by the National Institutes of Health. C57BL/6 J mice (Jackson Labs, Bar Harbor, ME) were allowed to acclimate in UAB rodent housing facility for 2 weeks before breeding. Males were removed from female cages after 17 days. Females were checked daily to determine the exact date of birth for pups, designated as postnatal day 0 (PD0). Litters were assigned randomly to MSEW or normally reared control groups for each breeding round. MSEW litters were separated from the dam for 4 h/day (900–1300 h) during PD2‐5, 8 h/day (900–1700 h) during PD6‐16, and weaned to clean cages on PD17 (George et al, 2010, Ho et al., 2016). During the MSEW protocol, pups were kept in an incubator (35°C, 60% humidity) for the duration of the separation time each day. Normally reared mice were left undisturbed with dams from birth until weaning at PD21 as previously described (George et al 2010, Ho et al., 2016). Normally reared mice are referred to as controls in the present study. Females were given a one‐week minimum rest period between breeding cycles. Mice were weighed at the time of termination for experimental endpoints with 3‐ to 6‐month‐old adult male mice (n= 46–50 per control and stress groups). Experiments included randomly assigned mice from 1 to 3 distinct dams and litters. This age and sex of normally reared and MSEW adult mice were studied to correlate with our previous publication detailing derangements in aortic function (Ho et al., 2016).

### 2.2 Hematologic analyses and hematocrit

MSEW and control mice (*n* = 5 per group) were briefly anesthetized using isoflurane and whole blood was collected via retro‐orbital bleed using heparinized micro‐hematocrit capillary tubes (Thermo Fisher Scientific Waltham, MA) for hematocrit analysis. Fresh samples were analyzed using a veterinary hematology system to determine hematocrit, and complete blood count parameters with differentials (Hemavet 950FS, Drew Scientific Group, Miami Lakes, FL).

### 2.3 Analyses of heme, hemin, hemopexin, haptoglobin and erythropoietin

Mice (*n* = 6–12 per group) were anesthetized using isoflurane and 0.1 ml Brevital^®^. Blood was collected with either EDTA or heparin via cardiac puncture and centrifuged 15 min at 2000 *g* to collect plasma. The plasma was flashed frozen and stored at –80°C. Plasma concentrations of heme (μM), hemin (μM), hemopexin (ng/ml), and haptoglobin (ng/ml, or mg/dl, or pg/ml) were measured using available kits following the manufacturer's directions (QuantiChrom™ Heme Assay Kit 75877–998, BioAssay Systems, Hayward, CA; Mouse Hemopexin ELISA Kit ab157716, Abcam, Cambridge, MA; Haptoglobin Mouse ELISA Kit ab157714, Abcam, Hemin Assay kit, ab65332, Abcam; Quantikine Mouse EPO Immunoassay, MEP00B R&D Systems, Minneapolis, MN).

### 2.4 Heme‐dependent superoxide production by mouse aortic endothelial cells

Mouse aortic endothelial cells (MAECs; Cell Biologics, Chicago, IL) of passages 9–10 were incubated at 37°C for 24 h in complete media (Endothelial Cell Medium with growth factor supplement, M1168; Cell Biologics, Chicago IL) containing plasma (25 μl/ml) from either control or MSEW mice. Superoxide production by the MAECs was assessed using the dihydroethidium (DHE) method (Fink et al., 2004; Ho et al., 2016). Briefly, after 24 hours of incubation with plasma, cells were washed and incubated with either PEG‐SOD (20 U/mL) or vehicle (dH_2_O) in Krebs HEPES Buffer (KHB; 99.01 mM NaCl, 4.69 mM KCl, 2.5 mM CaCl_2_,1.2 mM MgSO_4_, 25 mM NaHCO_3_, 1.03 mM K_2_HPO_4_, 20 mM Na‐HEPES, 5.6 mM dextrose; pH 7.35) for 1 h. This was followed by a 30‐min incubation with DHE (Life Technologies, Carlsbad, CA), after which the cells were rinsed and scraped in KHB. Samples were placed in methanol, stored at –80°C for 30 min, and then homogenized and filtered. Aliquots (200 μl) of each sample were pipetted into glass vials for HPLC analysis of oxyethidium (Elite LaChrome, Hitachi High‐Technologies, Tokyo, Japan). For Bradford protein assay, 50 μl of homogenate was added to 50 μl NaOH. To determine whether the heme pathway mediated activation of superoxide production, cells with plasma from control or MSEW mice were also incubated with either the heme scavenger hemopexin (0.25 µg/µL plasma; Athens Research and Technology, Athens, GA; 16–16–080513), the TLR4 antagonist TAK‐242 (1 µM; InvivoGen, San Diego, CA, tlrl‐cli95), or vehicle (PBS for hemopexin, 0.001% DMSO for TAK‐242). Superoxide production was calculated by subtracting the value obtained in PEG‐SOD‐treated samples from vehicle‐treated samples and reported as pmol oxyethidium/mg protein.

### 2.5 Tissue analyses of haptoglobin, hemopexin and heme oxygenase

Spleen (*n* = 7 per each group) and liver (*n* = 5–6 per group) were dissected from mice, flash frozen in liquid nitrogen, and stored at –80°C. Frozen spleen and liver from control and MSEW mice were homogenized using a glass‐on‐glass homogenization method as previously described (Ho et al., 2016) and ELISA kits were performed per the manufacturer's instructions (Mouse Hemopexin ELISA Kit ab157716, Abcam; Haptoglobin Mouse ELISA Kit ab157714, Abcam). Protein concentration was determined via Bradford assay (Bradford Protein Assay, Bio‐Rad, Hercules, CA). Splenic haptoglobin levels (ng/mg protein) and liver hemopexin (pg/mg protein) measurements were reported.

To determine heme oxygenase isoform levels by Western blot, frozen spleen samples (*n* = 7 per group) were homogenized (50 mg tissue/ml homogenization buffer) in buffer (10 mM Tris‐HCl, 140 mM NaCl, 5 mM EDTA, 1% Triton X‐100, 1% sodium deoxycholate, 0.1% SDS, 0.025% NaN_3_, 2 mM PMSF, 10 µM leupeptin, 2 µM pepstatin A, and 1 µg/ml aprotinin) via glass‐on‐glass homogenization. Samples were sonicated for 3 × 3‐sec bursts on ice and centrifuged at 17,000 *g* for 20 min at 4°C. Supernatant was collected and stored at –80°C. Samples were run on 12% SDS‐polyacrylamide gels, transferred on polyvinylidene difluoride membranes (Immobilon‐FL, MilliporeSigma, Burlington, MA), and incubated overnight at 4°C with antibodies against heme oxygenase‐1 (HO‐1; ab13243 Lot No. GR123437‐14, Abcam) or heme oxygenase‐2 (HO‐2; ab90492 Lot No. GR144478‐1, Abcam), as well as β‐actin (A1978, Sigma Aldrich, St. Louis, MO). Membranes were incubated with secondary antibodies goat‐anti‐rabbit 680 (35569, Invitrogen) or goat‐anti mouse 800 (2101150, Rockland Immunochemicals, Inc.; Limerick, PA) for 1 hr at room temperature in the dark. Blots were imaged using the Odyssey CLx Infrared Imaging System and bands were quantified for statistical analysis (Image Studio Software, LI‐COR Imaging Systems, Lincoln, NE, version 5.2). Results were normalized to β‐actin densitometry. Data are reported as fold‐change relative to control.

### 2.6 Tissue levels of TLR4 mRNA expression and TLR4 protein localization

#### 2.6.1 mRNA expression

Thoracic aortae (*n* = 6–8 per group) were isolated and cleaned of any adherent adipose tissue, flashed frozen in liquid nitrogen, and stored at –80°C. Frozen aortae from control and MSEW mice were homogenized in TRIzol (Invitrogen, Grand Island, NY) using a glass‐on‐glass homogenization method as previously described (Ho et al., 2016). RNA isolation was performed using TRIzol, per the manufacturer's instructions, and cDNA was synthesized using the QuantiTect Reverse Transcription Kit (Qiagen, Valencia, CA). Quantitative reverse transcriptase PCR was performed for the detection of *Tlr4* (QuantiTect Primer Assay Mm_Tlr4_1_SG, Qiagen, 249900) using the QuantiFast SYBR Green RT‐PCR Kit (Qiagen) and BioRad CFX96. The comparative method of relative quantification (2^− ΔΔCt^) was used to calculate the expression level of *Tlr4*, normalized to *Gapdh* (Mm_Gapdh_1_SG QuantiTect Primer Assay, Cat. No. QT00199388). Data are reported as a fold‐change from control mice.

#### 2.6.2 Histology

Thoracic aortae (*n* = 6–7 per group) were fixed in 1% neutral buffered formalin for 24 h at room temperature and dehydrated in an increasing series of ethanol prior to being embedded in paraffin blocks. The sections of aortae were cut at 5 μm. Mounted sections were blocked in 10% goat serum at room temperature for 20 min then washed with 10 mM PBS for 5 min. Sections were incubated with primary antibody anti‐rabbit TLR4 (Novis Biologicals; NB100‐56580; 1:500 dilution in PBS) at 4°C overnight and subsequently washed with 10 mM PBS for 5 min and incubated with Alexa Fluora 594 goat anti‐rabbit IgG (Molecular Probes; A11012; 1:2000 in PBS) at room temperature for 1 h. After washing slides with 10 mM PBS for 10 min (three times), slides were mounted with ProLong Diamond Antifade Mountant with DAPI (Invitrogen). An investigator blinded to the group assignments captured representative images of the aortic endothelium (at least 10 regions of interest per aorta) using CellsSens Software (Olympus, Center Valley, PA). The total integrated intensity was measured within the region of interest and divided by the total area (ImageJ v1.8). These values were averaged for each sample.

#### 2.6.3 Statistics

All data are expressed as mean ±SEM. Differences in superoxide production were assessed via a two‐way ANOVA with Sidak's multiple comparison test. All other data were analyzed using Student's *t*‐*test* with two‐tailed *p* value. A value of *p* ≤ 0.05 was considered statistically significant.

## 3 RESULTS

### 3.1 Exposure to MSEW induces increased mean red blood cell corpuscular hemoglobin concentration, increased hematocrit, and increased heme in adult mice

**FIGURE 1:**
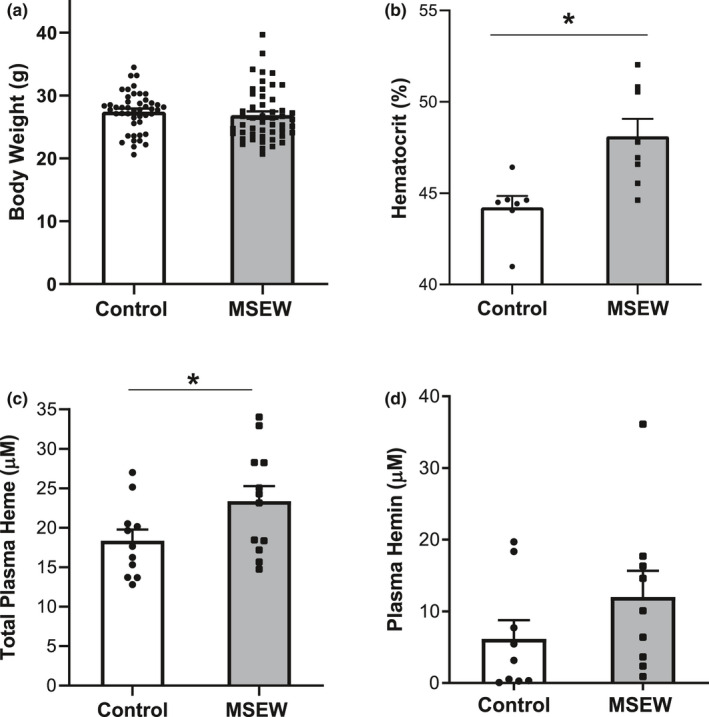
MSEW induces circulating heme. (a) body weights of mice utilized in this study (*n* = 46–50 per group: *p* = 0.45). (b) hematocrit measured in blood samples from anesthetized MSEW and control mice (*n* = 7–8 per group: *p = 0.006). (c) total plasma heme concentration in MSEW and control mice (*n* = 11–12 per group; *p = 0.0495). (d) plasma hemin concentration in MSEW and control mice (*n* = 9 per group; *p* = 0.21). Data analyzed in panel A‐D using Student's t test.

Previous studies with the MSEW mouse model have shown that exposure to MSEW does not affect weight gain in neonatal pups or in young adult mice (George et al., 2010, Ho et al., 2016). We confirmed that the mice utilized throughout this study, ages 3–6 months, also showed no difference in body weights (*p* = 0.45, Figure 1a). We performed hematologic analysis on blood samples from MSEW and control mice (*n* = 5 per group, Table 1). Hematocrit was significantly higher in MSEW mice compared to normally reared control mice (*p* = 0.039, Table 1). Hematocrit, measured using blood capillary tubes, was significantly higher in MSEW compared to control (*p* = 0.006, Figure 1b). Additionally, total hemoglobin levels were significantly higher in MSEW compared to control mice (*p* = 0.003, Table 1) and the mean corpuscular hemoglobin concentration (MCHC, denoting hemoglobin levels within individual red blood cells) was also significantly elevated (*p* = 0.01, Table 1). There was a tendency toward increased red cell hemoglobin content (*p* = 0.06, Table 1). Finally, the total lymphocyte count was significantly lower in MSEW mice compared to controls (*p* = 0.03, Table 1), while all other blood cell counts were similar in MSEW and control mice.

**TABLE 1:** Hematologic analysis of in MSEW and Control mice. Blood count parameters with differential in mice (*n* = 5 per group) detected with veterinary hematology system using Hemavet 950FS (Drew Scientific Group, Miami Lakes, FL). For all parameters, the mean, SEM, and *P*‐value are shown; statistically significant comparisons are bolded (K/μl: thousand per μl; M/μl: million per μl). Data analyzed using Student's t test.

Parameter	Control Mean ±SEM	MSEW Mean ±SEM	*p* value
Red Blood Cells (M/μl)	9.09 ± 0.23	9.40 ± 0.07	0.233
Hemoglobin (g/dl)	14.18 ± 0.26	15.46 ± 0.17	**0.003**
Hematocrit (%)	41.06 ± 0.79	43.38 ± 0.51	**0.039**
Mean Cell Volume (fl)	45.20 ± 0.73	46.16 ± 0.41	0.287
Mean Cell Hemoglobin (pg)	15.62 ± 0.34	16.44 ± 0.16	0.059
Mean Cell Hemoglobin Concentration (g/dl)	34.54 ± 0.24	35.66 ± 0.26	**0.012**
Red Blood Cell Distribution Width (%)	17.92 ± 0.28	17.28 ± 0.18	0.092
White Blood Cells (K/μl)	22.80 ± 1.54	20.02 ± 1.17	0.190
Neutrophils (K/μl)	5.07 ± 0.47	4.80 ± 0.61	0.727
Lymphocytes (K/μl)	15.08 ± 0.83	12.57 ± 0.49	**0.032**
Monocytes (K/μl)	1.39 ± 0.24	1.25 ± 0.14	0.614
Eosinophils (K/μl)	0.93 ± 0.19	1.02 ± 0.09	0.671
Basophils (K/μl)	0.33 ± 0.07	0.39 ± 0.04	0.484
Neutrophils (%)	22.16 ± 0.95	23.60 ± 1.81	0.501
Lymphocytes (%)	66.43 ± 1.74	63.10 ± 1.36	0.169
Monocytes (%)	6.03 ± 0.86	6.27 ± 0.67	0.834
Eosinophils (%)	3.98 ± 0.79	5.10 ± 0.27	0.214
Basophils (%)	1.40 ± 0.30	1.94 ± 0.16	0.156

The impact of ELS on circulating heme levels was determined in samples obtained from anesthetized mice. Total plasma heme was 27% higher in MSEW mice than in control mice (Figure 1c; *p* = 0.0495). Total plasma heme includes heme bound to protein and free heme (Belcher et al., 2017). Plasma levels of hemin, the oxidized Fe^3+^ state of unbound free heme, did not differ significantly between MSEW and controls (Figure 1d; *p* = 0.21).

### 3.2 MSEW mice demonstrate reduced splenic haptoglobin abundance, but not heme oxygenase or hemopexin

**FIGURE 2:**
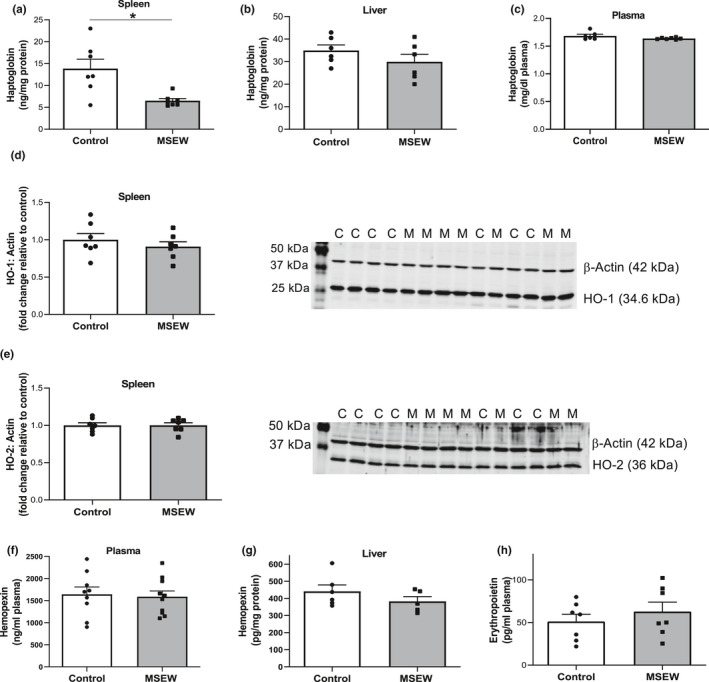
Splenic haptoglobin level is decreased in MSEW mice. Haptoglobin levels in (a) spleen (*n* = 7 per group; **p = 0.007), (b) liver (*n* = 6 per group; *p* = 0.26), and (c) plasma (*n* = 6–7 per group; *p* = 0.11) of control and MSEW mice. (d) *left*: quantification of western blot densitometry data of splenic heme oxygenase‐1 (HO‐1) normalized to β‐actin and reported as fold‐change relative to control; (d) *right*: representative western blot images of HO‐1 protein in control and MSEW mice (C and M, respectively; *n* = 7 per group; *p* = 0.39). (e) *left*: quantification of the western blot densitometry data of splenic heme oxygenase‐2 (HO‐2) normalized to β‐actin and reported as fold‐change relative to control. (e) *right*: representative western blot images of HO‐2 protein in control and MSEW mice (C and M, respectively; *n* = 7 per group; *p* = 0.99). (f) hemopexin levels in plasma from MSEW and control mice (*n* = 10 per group; *p* = 0.79). (g) hemopexin levels in liver from MSEW and control mice (*n* = 5 per group; *p* = 0.26). (h) erythropoietin levels in plasma from MSEW and control mice (*n* = 7 per group; *p* = 0.42). Data in panels A‐H analyzed using Student's t test.

We investigated whether heme degradation pathways were dysregulated in mice exposed to MSEW. Haptoglobin is primarily produced in the liver and binds hemoglobin leading to its clearance by the spleen, liver, and macrophages (Huntoon et al., 2008; Shih et al., 2014). Splenic haptoglobin levels in MSEW mice averaged only 53% of values observed in control mice (Figure 2a; *p* = 0.007). In contrast, haptoglobin concentrations in the liver (Figure 2b) and plasma (Figure 2c) did not differ significantly between MSEW and control mice. Heme oxygenase isoforms are proteins responsible for scavenging free heme and combatting oxidative stress (Elbirt & Bonkovsky, 1999; Thomsen et al., 2013). MSEW and control mice did not significantly differ with regard to abundance of HO‐1 or HO‐2 isoforms in the spleen (Figure 2d and 2e, respectively). Similarly, MSEW and control mice did not significantly differ with regard to endogenous concentrations of the free heme scavenger hemopexin in plasma (Figure 2f) or liver (Figure 2g). Erythropoietin (EPO) regulates heme and red blood cell biosynthesis (Chung et al., 2017). Circulating EPO levels showed no significant difference between MSEW and control mice (Figure 2h).

### 3.3 MSEW increases aortic Tlr4 expression and endothelial TLR4 abundance

**FIGURE 3:**
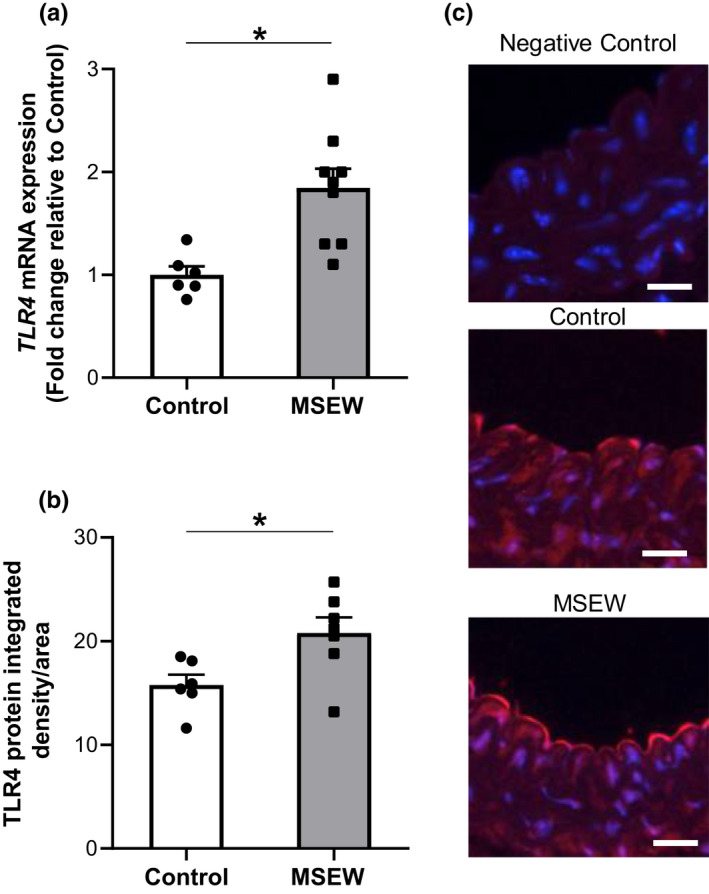
TLR4 expression and endothelial TLR4 abundance are increased in MSEW aortae. (a) *Tlr4* mRNA expression measured by qRT‐PCR (*n* = 6–9 per group, *p = 0.004). (b) TLR4 protein integrated density in the endothelium of aortae from control and MSEW mice measured via immunofluorescence (*n* = 6–7 per group, *p = 0.02). (c) representative images of immunofluorescence staining for TLR4 (red) in the thoracic aorta that are quantified in panel *B*. Nuclei are stained with DAPI (blue), Scale bar = 20 μm. Data in panels A‐C analyzed using Student's t test.

We determined the impact of ELS on TLR4 protein levels and mRNA expression in the thoracic aorta. We utilized the thoracic aorta similar to our previous work showing MSEW induces aortic endothelial dysfunction (Ho et al., 2016). In adulthood, *Tlr4* expression in the aorta of MSEW mice was nearly twice that of control mice (Figure 3a; *p* = 0.004). Immunofluorescence images revealed the TLR4 protein to be localized primarily in the endothelium (Figure 3c), with integrated density 32% greater in MSEW mice than in control mice (Figure 3b; *p* = 0.022).

### 3.4 Plasma from mice exposed to MSEW induces superoxide production

**FIGURE 4:**
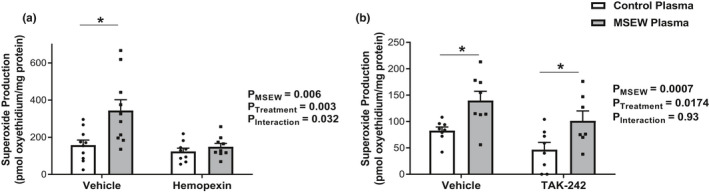
MSEW‐induced heme activates superoxide production in cultured MAECs. (a) Superoxide production by MAECs incubated with plasma from control mice (control plasma) or MSEW mice (MSEW plasma) and treated with either vehicle or hemopexin for 24 h (*n* = 9–10 per group). (b) Superoxide production by MAECs incubated with plasma from control mice (control plasma) or MSEW mice (MSEW plasma) and treated with either vehicle or TAK‐242 for 24 h (*n*= 7–8 per group). *P* values shown in panels *A* and *B* are main effects determined by 2‐way ANOVA, with the following results from Sidak's multiple comparison test: (a) control vs MSEW +vehicle: *p = 0.001; control vs MSEW +hemopexin: *p* = 0.87. (b) control vs MSEW +vehicle: *p = 0.02; control vs MSEW +TAK‐242: *p = 0.03.

To test the biological relevance of increased plasma heme levels in MSEW mice, superoxide production was measured in cultured mouse aortic endothelial cells (MAECs) incubated with plasma from MSEW mice or control mice. We incubated the plasma in the presence or absence of exogenous hemopexin (free heme scavenger). With vehicle treatment, incubation with MSEW plasma led to significantly greater superoxide production by MAECs compared to cells incubated with control mouse plasma (Figure 4a; *p* = 0.001); however in the presence of hemopexin, superoxide production did not differ between MAECs incubated with plasma from control vs MSEW mice (Figure 4a; *p* = 0.87). The results (Figure 4a) revealed a significant interaction between the effects of MSEW plasma and hemopexin treatment on superoxide production (two‐way ANOVA: *P*_interaction_ = 0.032). These data implicate the increased plasma levels of free heme as a major contributor to MSEW‐induced superoxide production.

To test whether the plasma from MSEW mice also activates the TLR4 system, cultured MAECs were incubated with plasma from MSEW or control mice, in the presence or absence of TAK‐242, a TLR4 antagonist. Accordingly, superoxide production by MAECs incubated with MSEW plasma was significantly greater than the control plasma group (Figure 4b), regardless of whether the cells were co‐incubated with vehicle (*p* = 0.02) or TAK‐242 (*p* = 0.03). Thus, in contrast with the effects of hemopexin, TAK‐242 treatment did not alter the increase in superoxide production with plasma from MSEW mice (Figure 4b). These data suggest that the activation of superoxide production in this *in vitro* system is independent of TLR4 activation but dependent on free heme.

## 4 DISCUSSION

The findings of this study support the hypothesis that exposure to MSEW in male mice leads to heme dysfunction and loss of heme homeostasis in adults. Specifically, adult mice with exposure to MSEW demonstrate: (a) increased hemoglobin, mean corpuscular hemoglobin concentration, and hematocrit; (b) reduced abundance of splenic haptoglobin; (c) increased *tlr4* mRNA expression and TLR4 abundance localized to the aortic endothelium; and, (d) increased circulating levels of heme, a known pro‐oxidant mediator. These results indicate that ELS leads to elevation of circulating heme, possibly through dysregulation of hemoglobin clearance from the reduction in splenic haptoglobin. Previous reports show that exposure to MSEW in male mice leads to endothelial dysfunction in adulthood (Ho et al., 2016). Thus, we propose that ELS induces a dysfunctional heme pathway as a potential mediator of endothelial dysfunction and greater CVD risk in adulthood.

Stress early in life, either experienced by humans or in animal models, leads to CVD risk and a putative mechanism is through increased pro‐oxidants in adulthood (Obi et al., 2019). The findings of the current study demonstrate that ELS induces heme/hemoglobin dysregulation. Although measurements of free heme using commercially available kits are controversial (Aich et al., 2015; Belcher et al., 2017; Oh et al., 2016), we validated elevated heme levels in plasma from MSEW mice with a hemopexin‐dependent *in vitro* assay providing a potential mechanistic pathway for pro‐oxidant activation. Hemopexin binds extracellular heme with a very high affinity (Hvidberg et al., 2005). In a model of heme overload, hemopexin treatment limited ROS production (Vinchi et al., 2013). Thus, the results of our *in vitro* assay support the concept that ELS exposure increases circulating free heme and are consistent with previous studies that circulating heme has pro‐oxidant effects (Jeney et al., 2002). Excess free heme promotes the generation of ROS (Duvigneau et al., 2019; Vasconcellos et al., 2016), endothelial dysfunction (Gladwin & Kato, 2007), and has the potential to oxidize low‐density lipoprotein (LDL), a process known to promote atherosclerosis (Balla et al., 1991). Previously, our group reported that ELS exposure induces endothelial dysfunction through increased superoxide production in the aorta (Ho et al., 2016) of adult male mice similar to the mice included in this present study. Interestingly, Straub's and Isakson's laboratories previously reported that vascular hemoglobin levels modulate NO bioavailability and redox homeostasis leading to functional regulation of vasoconstriction and vasorelaxation (Galley & Straub, 2017; Straub et al., ,^2012^, ^2014^). Thus, our new findings showing that exposure to MSEW induces excess circulating hemoglobin and heme may also point towards increased vascular hemoglobin levels resulting in a loss of NO bioavailability within the vascular wall as well as in the circulation.

Excess free heme and hemoglobin have been shown to be detrimental, inducing red blood cell hemolysis, cytokine upregulation, inflammation, and ROS production via TLR4‐independent and ‐dependent pathways (Belcher et al., 2014; Gladwin & Kato, 2007; Graca‐Souza et al., 2002; Janciauskiene et al., 2020; Porto et al., 2007; Sawicki et al., 2015). The deleterious effects of elevated heme in the body are mitigated via scavenging mechanisms and homeostatic regulation. Heme is predominately synthesized in the mitochondria, and only small amounts of free heme are usually present in the bloodstream; most heme is bound in protein complexes such as hemoglobin (Sawicki et al., 2015; Wagener et al., 2003). Haptoglobin is primarily produced in the liver and is important for binding free hemoglobin from lysed red blood cells *in vivo* (Shih et al., 2014). Haptoglobin facilitates the uptake of hemoglobin by CD163 on macrophages preventing heme‐mediated injury (Galicia et al., 2009; Lim et al., 1998; Thomsen et al., 2013). Low levels of circulating haptoglobin reflects an active phase of hemolysis (Barcellini & Fattizzo, 2015; Shih et al., 2014). In the present study, MSEW and control mice showed similar levels of circulating and liver‐derived haptoglobin levels suggesting that there is not ongoing hemolysis. The spleen is also critical for hemoglobin clearance (Schaer et al., 2014). We found that MSEW mice displayed an approximately 50% reduction in splenic haptoglobin levels compared to control mice. Lower splenic haptoglobin levels may reflect decreased levels of the hemoglobin‐haptoglobin system (Schaer et al., 2014), and dysfunctional splenic hemoglobin clearance. The present study also showed that exposure to MSEW leads to increased levels of total hemoglobin compared to controls. The elevated hemoglobin is associated with a higher hemoglobin concentration within red blood cells but not with a significantly higher number of red blood cells. This finding indicates that ELS exposure may increase hemoglobin synthesis during hematopoiesis which may also be an upstream mechanism contributing to higher heme levels in MSEW mice. We also observed higher hematocrit in blood samples from MSEW mice, without higher numbers of red blood cells or increased cell volume, which would implicate lower plasma volume in mice exposed to MSEW. We previously reported that MSEW does not lead to any changes in food or water intake (Ho et al., 2016), thus further in‐depth analysis is needed to examine whether MSEW leads to a resetting of the homeostatic mechanisms governing volume status in mice.

The heme‐hemopexin pathway may function in conjunction with, or as an alternate pathway to, the hemoglobin‐haptoglobin pathway (Smith & McCulloh, 2015). Hemopexin is mainly produced in the liver and is released into circulation where it binds free heme to protect against heme‐mediated oxidative stress (Morello et al., 2009). We failed to detect a significant difference in hemopexin levels in the liver or in the plasma between control and MSEW mice, indicating that hemopexin likely does not play a role in the increased free heme observed in this model. Once intracellular, heme is broken down into carbon monoxide, iron, and biliverdin by heme oxygenase (HO; HO‐1 and HO‐2) (Maines, 1988; Thomsen et al., 2013). HO‐1 is a ubiquitous inducible cellular stress protein exerting a major role in cellular defense mechanisms (Ryter et al., 2006; Ryter & Choi, 2009). HO‐2 is highly expressed in different tissues such as testis and smooth muscle cells from cerebral vessels (Muñoz‐Sánchez & Chánez‐Cárdenas, 2014). There is novel evidence suggesting HO‐2 plays a protective role in cellular damage and importantly in the regulation of O_2_‐sensing systems (Muñoz‐Sánchez & Chánez‐Cárdenas, 2014). We observed no differences in HO‐1 or HO‐2 protein abundance; however, we did not determine HO enzyme activity or cellular localization, which is a limitation to our study. Further work is necessary to delineate these potential roles in MSEW, although in general our findings suggest that the induction of heme with ELS exposure is not due to a disruption in heme metabolism.

TLR4 is part of a larger family of toll‐like receptors, which together form a subset of pattern recognition receptors of the innate immune system. Pattern recognition receptors recognize conserved pathogen‐associated molecular patterns and damage‐associated molecular patterns and are responsible for early activation of the innate immune system in response to infection and tissue injury (Mogensen, 2009). Exaggerated TLR4 signaling is implicated in the progression of numerous pathological CVD states, including hypertension, atherosclerosis and heart failure (Hernanz et al., 2015; Timmers et al., 2008; Yu & Feng, 2018). Endothelium‐specific TLR4 activation was shown to be a critical mediator of stroke (Tang et al., 2017). A study using a murine sickle cell disease model showed that heme‐mediated TLR4 signaling activates vaso‐occlusion (Belcher et al., 2014). These studies support the hypothesis that enhanced endothelial TLR4 abundance is a risk determinant of CVD. Moreover, TLR4 activation has been implicated in depressive and anxiety behavior disorders (Garcia Bueno et al., 2016; Habib et al., 2015; Strekalova et al., 2015), which are also at risk with exposure to ELS. ELS‐mediated activation of TLR4 in rodents has been implicated in reprogramming of neural anti‐inflammatory pathways and also results in increased anxiety behavior during adulthood (Mouihate et al., 2010; Sominsky et al., 2013). The results of this current study show that MSEW exposure increases *Tlr4* mRNA expression and TLR4 protein abundance localized to the aortic endothelium in mice. Further work is necessary to understand the interactions of heme and TLR4 in this model. Regardless, increased TLR4 levels in the endothelium suggests potential deleterious effects of ELS on CVD through non‐heme TLR4‐dependent processes.

Erythropoiesis is a homeostatic process in the bone marrow producing new erythrocytes with the turnover of senescent erythrocytes by macrophages in the spleen. In times of erythroid demand such as tissue hypoxia, stress erythropoiesis is invoked and becomes the predominate mechanism until steady‐state resumes (Paulson et al., 2020). Stress erythropoiesis is localized mainly in the liver and spleen (Bennett et al., 2019). Interestingly, studies have demonstrated stress erythropoiesis is activated after chronic restraint stress, a mouse model of chronic psychological stress (Vignjevic Petrinovic et al., 2020; Vignjević et al., 2014). Voorhees et al showed in chronic restraint stress that stress erythropoiesis was activated through induced glucocorticoid signaling in the absence of elevated EPO levels (Voorhees et al., 2013). Recently, Bennett et al showed that inflammation induces stress erythropoiesis through a heme‐dependent mechanism and TLR signaling (Bennett et al., 2019). Thus, we propose that exposure to ELS may induce inflammation, increased heme, and a state of stress erythropoiesis. Lower lymphocyte numbers is indicative of a state of stress erythropoiesis. While we did not directly characterize stress erythropoiesis in the current study, this represents an interesting theory potentially linking psychological stress, hematologic and heme derangements, and enhanced TLR signaling similar to what we observed with this model of ELS.

In conclusion, our findings provide evidence that exposure to ELS leads to dysregulation of the heme pathway and upregulation of TLR4 in the aortic endothelium. Further, we demonstrate that plasma from MSEW mice enhances endothelial superoxide production via increased free heme. We previously demonstrated that MSEW mice have superoxide‐dependent endothelial dysfunction; here, we argue that the induction of the heme pathway may be an upstream mechanism of this effect. Thus, exposure to ELS may contribute to CVD risk through maladaptive processes involving hematologic derangements, inflammation, oxidative stress, and endothelial dysfunction.

## AUTHOR CONTRIBUTIONS

D.H.H., T.H.N., and J.S.P. designed experiments; Y.D.P., D.H.H, J.C., M.B., J.C., X.L., B.M.F., and K.A.H. performed experiments; Y.D.P., T.H.N., D.H.H., C.E.K., K.A.H., and J.S.P. analyzed data; Y.D.P, D.H.H, T.H.N., C.E.K., K.A.H., and J.S.P. interpreted data; Y.D.P., T.H.N., C.E.K., K.A.H., and J.S.P. prepared figures; Y.D.P., T.H.N., and J.S.P. drafted the manuscript; all authors approved the final version of the manuscript.
